# Oral Administration of Polymer Hyaluronic Acid Alleviates Symptoms of Knee Osteoarthritis: A Double-Blind, Placebo-Controlled Study over a 12-Month Period

**DOI:** 10.1100/2012/167928

**Published:** 2012-11-20

**Authors:** Toshiyuki Tashiro, Satoshi Seino, Toshihide Sato, Ryosuke Matsuoka, Yasunobu Masuda, Naoshi Fukui

**Affiliations:** ^1^Department of Orthopaedic Surgery, JR Tokyo General Hospital, 2-1-3 Yoyogi, Shibuya-ku 151-8528, Tokyo, Japan; ^2^Research and Development Division, Kewpie Corporation, 5-13-1 Sumiyoshi-cho, Fuchu 183-0034, Tokyo, Japan; ^3^Department of Life Science, Graduate School of Arts and Sciences, The University of Tokyo, 3-8-1 Komaba, Meguro-ku 153-8902, Tokyo, Japan

## Abstract

This study was conducted to investigate the efficacy of oral hyaluronic acid (HA) administration for osteoarthritis (OA) in knee joints. Sixty osteoarthritic subjects (Kellgren-Lawrence grade 2 or 3) were randomly assigned to the HA or placebo group. The subjects in the HA group were given 200 mg of HA once a day everyday for 12 months, while the subjects in the placebo group were given placebo. The subjects in both groups were requested to conduct quadriceps strengthening exercise everyday as part of the treatment. The subjects' symptoms were evaluated by the Japanese Knee Osteoarthritis Measure (JKOM) score. The symptoms of the subjects as determined by the JKOM score improved with time in both the HA and placebo groups. This improvement tended to be more obvious with the HA group, and this trend was more obvious with the subjects aged 70 years or less. For these relatively younger subjects, the JKOM score was significantly better than the one for the placebo group at the 2nd and 4th months after the initiation of administration. Oral administration of HA may improve the symptoms of knee OA in patients aged 70 years or younger when combined with the quadriceps strengthening exercise.

## 1. Introduction

Osteoarthritis (OA) is a disease characterised by a gradual loss of cartilage which extends over years and decades. Primary symptoms of the disease are pain and tenderness over the joint [1]. Although OA can affect any synovial joint, OA of knee joints is the most cumbersome in terms of prevalence and disability [2, 3]. OA is an age-related disease [4]. Therefore, the number of patients suffering from it is increasing in developed countries along with the aging of those societies. In the United States, for example, the number of patients with knee OA was 43 million in 1997 and is anticipated to be more than 60 million by 2020 [5]. Therefore, there is now a mounting demand for effective treatments for the disease. Considering the number and age of the patients with knee OA, such treatments should be safe and economically affordable. 

Hyaluronic acid (HA) is a mucopolysaccharide comprised of tandem repeats of D-glucuronic acid and N-acetyl glucosamine. It is abundantly present in the synovial fluid [6]. At present, intra-articular administration of HA is the treatment of choice for patients with symptomatic knee OA [7]. Compared with steroids, HA placed into joints may work more slowly, but its effect may last considerably longer [8]. According to the current protocol, HA should be administered repeatedly into the joint cavity [9]. This necessity for multiple injections is a major drawback of the therapy. To be treated with HA, patients must visit clinics repeatedly and must undergo the discomfort associated with the injections. Again, the risk of complication increases by the repetition of injections [10]. Considering these overt and potential disadvantages, it is far more desirable for the symptoms of knee OA to be relieved by oral administration of HA. 

Previous studies have suggested that the symptoms of knee OA might indeed be alleviated by HA ingestion [11–14]. When knee OA patients were treated with 240 mg of HA per day for 12 weeks, both the Japanese Knee Osteoarthritis Measure (JKOM) score and the Japan Orthopaedic Association (JOA) score improved significantly from levels before the treatment [11]. In another study, patients receiving oral administration of 200 mg of HA everyday for 8 weeks had significantly improved according to Western Ontario and McMaster University Osteoarthritis Index (WOMAC) scores compared with the patients given placebo [12]. Again, oral administration of 240 mg of HA per day for 8 weeks significantly improved the JKOM score [13]. In this study, the improvement was most obvious in the subscale for “pain and stiffness” of that score system. It was also reported that the JOA score of knee OA patients was significantly improved by the daily ingestion of 60 mg of HA for 16 weeks [14]. In this study, the improvement was more obvious with patients who performed therapeutic exercises in combination with HA therapy. Meanwhile, when 80 mg of HA was ingested daily for 8 weeks, physical function and total symptoms subscales of the WOMAC scores improved more apparently in the HA group compared with the control group, as did the role-physical and bodily pain subscales of the Short Form-36 Version 2 (SF-36v2) [15].

Although those studies report positive effects of orally administered HA on knee OA, they have certain study limitations such as the lack of appropriate controls or the shortness of study period. Therefore, it is still unclear whether orally administered HA indeed improves the symptoms of knee OA patients. To clarify this issue, we conducted a one-year, placebo-controlled double-blind study involving subjects with symptomatic knee OA.

## 2. Materials and Methods

### 2.1. Subjects

 A prospective, randomised, double-blind, placebo-controlled study was conducted to assess the efficacy of orally administered HA for symptom relief in older adults with knee OA. The study was performed at the JR Tokyo General Hospital between December 2009 and November 2010. The subjects were 60 Japanese male and female patients with symptomatic knee OA. They were recruited from among those who visited orthopaedic clinics of the institute, seeking treatment for their knee symptoms. To be included in the study, patients had to be 50 years of age or older and had to have OA of Kellgren-Lawrence (K/L) grade 2 or grade 3 [16] in at least one knee joint, which must have been symptomatic for at least 2 months prior to enrollment. If a subject had pain in both knees, the more painful knee had to meet these criteria. Patients with rheumatoid arthritis or any other inflammatory joint disease, or secondary OA, were excluded. Also, patients with any significant problems with liver, kidney, or their motor or nerve systems were not included in the study. If a subject was taking any supplements at the time of enrollment that might relieve knee symptoms, he or she was requested to discontinue their use at least one month prior to HA administration. Again, subjects who had had any intra-articular injections had to wait at least 3 months until the medication was initiated.

This study was conducted in accordance with the principles of the Declaration of Helsinki. The trial was conducted under the approval of the Ethical Committee of JR Tokyo General Hospital, and informed consent was obtained in writing from each subject prior to enrollment in the study. 

### 2.2. Hyaluronic Acid

The hyaluronic acid used in this study was manufactured by kewpie Corporation (Tokyo) (Hyabest (J)). This HA had been shown to have a purity of 97% and a molecular weight of approximately 900,000, as determined by HPLC. The HA was mixed with an appropriate amount of cornstarch (Cornstarch IPW, Nihon Shokuhin Kako Co., Ltd., Tokyo) and was administered in a form of hard capsules, each containing 50 mg of HA. The capsules were manufactured by Aliment Industry Co., Ltd. (Tokyo). 

### 2.3. Study Design

The study was conducted as a randomised double-blind placebo-controlled trial. The 60 subjects enrolled in the study were assigned to either the HA or the placebo group, each consisting of 30 subjects by means of stratified randomisation to equalize age, sex ratio, and K/L grade between the groups. Subjects in the HA group were requested to ingest 4 hard capsules containing a total of 200 mg of HA once a day after breakfast, everyday for 12 months. In the placebo group, subjects were asked to take 4 hard capsules which contained only cornstarch.

All subjects in this study were directed to conduct a quadriceps strengthening exercise with straight leg raising, which had been shown to be an effective therapy for knee OA [17]. The strengthening exercise was implemented in this study because a previous study reported that orally administered HA improved knee OA symptoms more obviously with the subjects who performed this exercise [14]. During the study period, subjects were asked to keep a daily log as proof of having performed this exercise daily. When a subject complained of severe knee pain, nonsteroidal anti-inflammatory drugs were administered orally or externally. HA was administered intra-articularly if a subject's pain was very severe. 

### 2.4. Evaluation of Therapeutic Effects of HA Ingestion

In this study, patients' symptoms were evaluated by the Japanese Knee Osteoarthritis Measures (JKOM) score, which is a score system developed for people with knee OA living a Japanese cultural lifestyle [18]. This score consists of 25 items that cover four different categories: “pain and stiffness,” “conditions in daily life,” “general activities,” and “health conditions.” An overall result was assessed by summing the scores from the 25 items, with results ranging from 25 (no complaint) to 125 (most severe condition possible). The symptoms of the subjects were recorded immediately before and at 2, 4, 6, and 12 months after the initial capsule administration, together with records of adherence to the medication and quadriceps exercise. In order to evaluate the progression of OA during the study period, anteroposterior knee radiographs were obtained in a weight-bearing position with a knee in an extended position twice, at the time of enrollment and at the end of the 12-month study period. On these radiographs, the severity of OA was evaluated by K/L grading. 

### 2.5. Statistical Analysis

Mann-Whitney *U* test was used to compare the ratios of JKOM scores relative to levels before the administration, between the HA and placebo groups at respective time points. Wilcoxon signed-rank test was used to compare the JKOM scores before treatment and at respective time points during the study. Chi-square test was used to determine the significance of the changes in the K/L grade and the treatments that the subjects required besides HA ingestion and the quadriceps strengthening exercise. Statistical analyses were conducted using Dr. SPSS for Windows II (SPSS Inc., Tokyo, Japan) with the level of statistical significance set at 0.05.

## 3. Results

### 3.1. Background of Subjects

Demographic and clinical characteristics of the subjects in the HA and placebo groups are shown in Table 1. At baseline, no significant difference was observed between the two groups with respect to age, sex ratio, JKOM score, K/L grade, or the use of additional medication. By the end of the study, 12 subjects in the HA group and 10 subjects in the placebo group were excluded from the analysis, and thus, the analysis was performed with the 18 remaining subjects in the HA group and the 20 subjects in the placebo group. For the subjects excluded from the analysis, the reasons for exclusion were as follows: withdrawal of consent, 4 and 2 subjects in the HA and placebo groups, respectively; occurrence of possible side effects, 4 and 2, respectively; poor adherence to the medication or the quadriceps strengthening exercise, 4 and 6, respectively. Possible side effects in the HA group were gastric discomfort in 2 subjects, reflux esophagitis in 1, and appetite loss in another. In the placebo group, skin irritation (itching or skin redness) was found in 2 subjects as a possible side effect. The frequencies of these problems were not statistically significant between the HA and placebo groups. Again, none of these problems was demonstrated to indeed be caused by the ingestion of HA or placebo. Regarding compliance with medication, subjects whose ingestion rate was below 75% were excluded from the analysis. Also, subjects with a low implementation rate of the quadriceps exercise (less than 60%) were not included in the analysis. For the remaining subjects, the average rate of ingestion was 95% ± 2% and 98% ± 1% in the HA and placebo groups, respectively, and the implementation rate of the exercise was 90% ± 12% and 90% ± 3% in the respective groups. 

### 3.2. JKOM Scores of All Subjects

The JKOM scores of subjects in the HA and placebo groups before and after HA administration are shown in Table 2. Though not significant, there was some difference in the JKOM scores at baseline between the HA and placebo groups (Table 1). Considering this difference, the change in JKOM scores after the initiation of administration was evaluated in each group by the relative ratio of the score (%) to that at baseline. When compared with the score at baseline, total JKOM scores reduced (improved) significantly at 2 months through 12 months, similarly in both the HA and placebo groups. Although the ratio was lower with the HA group at all four time points after the initiation of administration, the difference between the groups was not significant at any time point. 

Next, changes in the score were analysed in each of the four subscales that compose the JKOM score. The scores for “pain and stiffness in the knees” and “condition of daily life” subscales were significantly reduced from those at baseline at the 2nd month and later in both the HA and placebo groups. Again, the scores for “general activities” were significantly reduced from those at baseline at the 4th month and later in both groups. For those 3 subscales, scores for the HA group tended to be lower (better) than those for the placebo group, but the difference among the groups was not significant at any time point. Meanwhile, for the “health conditions” subscale, the score of the HA group was significantly lower than that of the placebo group at all 4 time points after the initiation of administration.

### 3.3. Kellgren-Lawrence Grade

The K/L grade of the subject did not change significantly in either the HA or placebo group over the 12-month study period (Table 3).

### 3.4. Additional Medication

No significant differences were noted between the HA and the placebo groups with regard to the number of subjects with oral or external use of NSAIDS, or intra-articular HA administration (Table 4).

### 3.5. JKOM Scores of Subjects Aged 50 to 70 Years

The symptoms of knee OA are known to aggravate with age 22. Thus, it may be possible that the effect of HA ingestion differs depending on the age of the subject. We divided the subjects into two groups: those over 70 years; 70 years or less and analysed the effect of HA treatment in those groups. 

**Figure 1:**
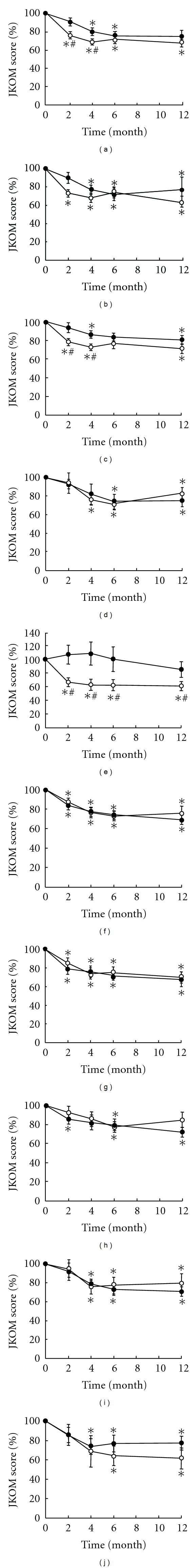
Change in JKOM score with respect to subject's age. Subjects were divided into two groups of younger (≦70 years of age; 21 subjects) and older subjects (older than 70 years of age; 17 subjects), and change of JKOM score was evaluated in the respective groups. For younger subjects, change rate of total score relative to that at baseline (a) is shown together with rates for “pain and stiffness in the knees” (b), “condition of daily life” (c), “general activities” (d), and “health conditions” subscales (e). Change rates of total JKOM score and those of 4 subscales for older subjects are shown in the same manner (f–j, resp.). Open and closed circles indicate change rates of subjects given HA and placebo, respectively. Results are mean ± SE of 7 to 11 subjects. *P < 0.05 against baseline and ^#^ *P* < 0.05 against placebo group.

Twenty-one subjects aged 70 years or less were assigned to the younger subject group. There were 6 males and 15 females in this group, and their mean age was 63.6 ± 0.9 years. For these subjects, the total JKOM score tended to reduce after the initiation of administration, as was observed for subjects of all ages. The reduction in the score was more obvious in the HA group, and thus the score for the HA group was significantly lower than that of the placebo group at 2 and 4 months after the initiation of administration (Figure 1(a)). 

Next, changes in score were analysed for each of the 4 subscales of the JKOM score. For the “pain and stiffness in the knees” subscale, the score reduced after beginning administration (Figure 1(b)). The reduction tended to be more obvious with the HA group, but the difference between the HA and placebo groups was not significant at any time point. The score for the “condition of daily life” subscale also reduced after the initiation of medication (Figure 1(c)). The reduction was more obvious with the HA group in the earlier period of administration, and thus the score of the HA group was significantly lower than that of the placebo group at the 2nd and 4th months. For the “general activities” subscale, again, the score reduced gradually after the administration was started (Figure 1(d)). The decline observed was equal between the HA and placebo groups, and thus the score differed little between them at any time point during the study period. For the last “health conditions” subscale, the change of the score after administration clearly differed between the HA and placebo groups (Figure 1(e)). For this subscale, the score of the HA group decreased soon after the initiation of administration, whereas the score in the placebo group changed little, or rather increased, after administration. Thus, the difference in the rate of change was statistically significant between the two groups at all four time points. 

### 3.6. JKOM Scores for Subjects 71 Years or Older

Of the 31 subjects included in the analysis, 17 were aged 71 years or older and were assigned to the older subject group. The group included 3 males and 14 females, and their mean age was 74.9 ± 1.5 years. For these subjects, again, the total JKOM score improved gradually after the initiation of administration (Figure 1(f)). The decline in scores occurred equally in the HA and placebo groups, and little difference was found with the score between the groups. 

Analysis of the changes of scores in the respective subscales revealed that in this age group, scores changed almost equally for HA and placebo groups. For “pain and stiffness in the knees,” “condition of daily life,” and “general activities” subscales, change of the score after administration was quite similar between HA and placebo groups, and little difference was observed with scores between the groups at any of the 4 time points (Figures 1(g)–1(i), resp.). For the last “health conditions” subscale, the change of the score after administration was quite similar for both groups up to the 4th month. At the 6th month and afterwards, the score for the placebo group tended to increase, while the score for the HA group remained low throughout the study period (Figure 1(j)). In this older subject group, the difference in scores was not significant between the HA and placebo groups in any subscale at any time point throughout the study period.

### 3.7. JKOM Scores and Radiographic Disease Severity

**Figure 2:**
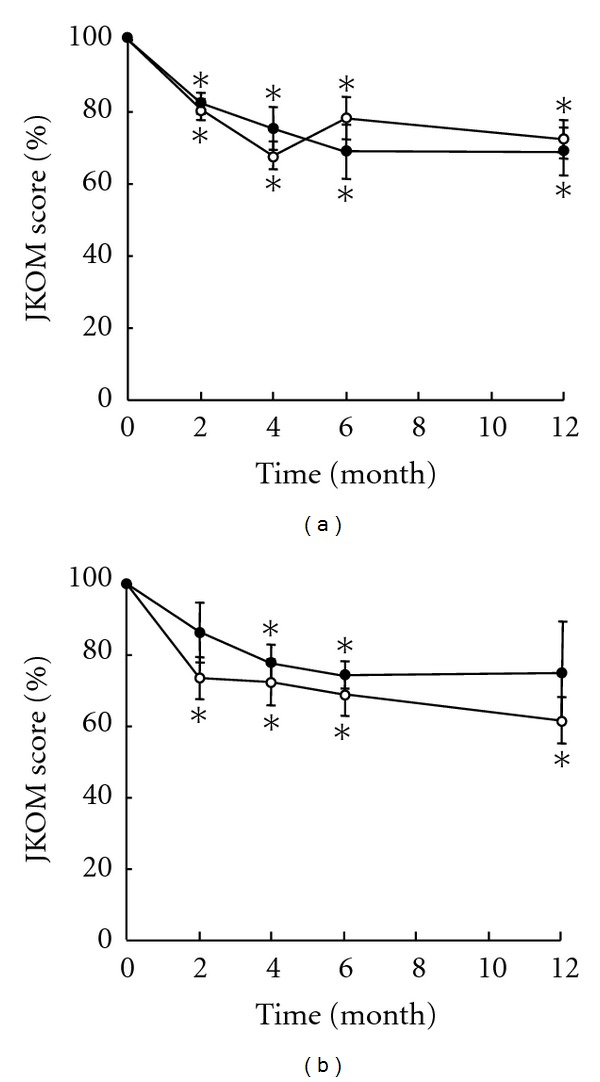
Change in JKOM score with respect to disease severity. Subjects were divided by the Kellgrel-Lawrence (K/L) grade determined on radiographs, and changes in the total JKOM score are shown in respective groups by change rates relative to scores at baseline. Results of subjects with K/L grade 2 knee OA (A) and those with K/L grade 3 OA (B) are shown. Open and closed circles indicate change rates of subjects given HA and placebo, respectively. Results are mean ± SE of 9 to 11 subjects. *P < 0.05 against baseline.

Finally, the effect of HA ingestion was evaluated in terms of disease severity on radiographs. In this analysis, the subjects were divided into two groups by K/L grade or radiographic severity of knee OA, and the change of the total JKOM score was investigated in respective groups. For 18 subjects with K/L grade 2 knee OA, the total JKOM score reduced significantly after the initiation of administration for HA and placebo groups alike (Figure 2(a)). Thus, the total JKOM score did not differ obviously between those administered HA and those given a placebo. Meanwhile, for 20 subjects with K/L grade 3 knee OA, reduction of the total JKOM score after administration tended to be more obvious in subjects who ingested HA, compared with those who took a placebo at all 4 time points, although the difference was not significant throughout the study period (Figure 2(b)).

## 4. Discussion 

In the present study, HA was administered orally. Therefore, it is crucial that the orally administered HA is indeed absorbed and distributed to the knee joints while retaining its biological activities. The results of animal experiments clearly demonstrated that this is possible. A series of experiments using radiolabelled HA indicated that orally administered HA would be indeed absorbed and distributed to the skin, bone, and synovial joints (including knee joints) and would be retained in those tissues for prolonged periods [19]. Orally administered HA may be degraded by bacteria in the intestine [20]. However, in the animal study, the pattern of distribution within the body and the time-course of clearance from the tissues indicated that a substantial part of orally administered HA should be absorbed without substantial degradation. In agreement with these findings, the results of clinical trials in humans showed that orally given HA is likely absorbed and distributed to the skin while preserving its biological activities [21, 22]. 

Therapeutic effects of HA on knee OA patients may not necessarily require the absorption of HA. A recent study reported that HA of a high molecular weight may bind to Toll-like receptor 4 (TLR4) and exert biological activities [23]. In this study, the association of HA with TLR4 was shown to increase the secretion of suppressor of cytokine signalling 3 (SOCS3), which leads to the suppression of proinflammatory cytokine expression. The binding of HA to TLR4 also suppresses the expression of pleiotrophin, which again contributes to the suppression of inflammation. Thus, the therapeutic effects of HA observed in this study may have been obtained by this mechanism, with the HA remaining in the intestines without absorption. 

There is another possibility that the therapeutic effect of HA could be obtained by mechanisms similar to glucosamine's. Glucosamine is another supplement which may alleviate symptoms of knee OA and can inhibit the progression of the disease [24–27]. Although its mechanism is not fully understood, glucosamine is considered to improve OA symptoms and inhibit disease progression by exhibiting chondroprotective and anti-inflammatory activities [28–32]. *In vivo*, glucosamine is converted to N-acetyl glucosamine within cells by the actions of a series of lysosomal enzymes [33]. As mentioned earlier, N-acetyl glucosamine is the monosaccharide that comprises HA together with D-glucuronic acid. Therefore, it is possible that N-acetyl glucosamine released from orally given HA may improve knee OA symptoms in the same manner as glucosamine. In support of this view, HA is reported to have an anti-inflammatory action analogous to glucosamine's [34]. 

As mentioned earlier, several studies have already shown the beneficial effects of orally administered HA on knee OA patients [11–15]. However, these studies tested the effect of HA over relatively short periods. Considering that knee OA is a disease that progresses gradually over an extended period of time that ranges from years to decades, and that the symptoms of knee OA fluctuate with time during the course of the disease [35], the effect of HA treatment should be evaluated over a longer period of time. This was our rationale for conducting this study.

The result of this study in which HA was administered orally over a 12-month period revealed a possibility that HA may alleviate knee OA symptoms in relatively young subjects in the early phase of the study period. In our study, the subjects' symptoms improved gradually during the study period in both the HA and placebo groups, as observed in previous studies [14, 15, 36]. When the results of all participating subjects were analyzed, the improvement of the total JKOM score tended to be more obvious in the HA group than in the placebo group, but the difference between the groups was not significant (Table 2). Then we divided the subjects by age and found that the younger subjects aged 70 years or less responded well to HA ingestion, while the treatment was less effective for the older subjects (Figure 1). For the younger subjects, the difference in JKOM scores between the HA and placebo groups was significant at the early time points (2nd and 4th months), but became insignificant later on (6th and 12th months). This apparent loss of the effect of HA treatment in the later phase might be ascribed to the improvement (decrease) of the score in the placebo group. That is, for the younger subjects, the effect of HA was not obvious at the later period possibly because the subjects were less symptomatic at that phase. If this view is correct, it may be right to say that the HA treatment tested in this study may be effective on a knee OA patient who is relatively young but symptomatic. Meanwhile, at present, we are unable to explain why the orally given HA was effective only on the younger subjects. Though merely speculative, the difference in effect by age could be ascribed to differences in cell metabolism. Alternatively, there may be some difference in the pathology of OA between the younger and older subjects. Obviously, further studies are necessary to elucidate the mechanism(s) that may account for the difference in the effect by age.

In this study, we performed another analysis to investigate whether response to the HA treatment differed by the severity of OA. In this analysis, subjects were divided by the K/L grade of knee OA, and the effect of HA administration was evaluated in the respective groups of subjects. Interestingly, the effect of HA treatment was not well observed in either subject group. This result might indicate that the pathology of OA may not differ much between K/L grade 2 and grade 3. Although not investigated in this study, it seems possible that the effect of HA treatment differs significantly between subjects with K/L grade 1 OA and those with grade 4 OA. At present, we are planning to conduct another study to clarify this point, recruiting a larger number of subjects. Hopefully, the result of this study will reveal the difference in the effect of orally given HA by the severity of the disease.

In the current trial, the administration of HA was combined with quadriceps strengthening exercise based on the result of a previous study [14]. The quadriceps exercise itself has been shown to be an effective therapy for symptomatic knee OA [17]. A study using MRI suggests that the increase in glycosaminoglycan (GAG) content within cartilage could be a possible mechanism for the efficacy of the treatment [37]. HA is a major structural component of articular cartilage. Within cartilage, HA serves to retain aggrecan within the cartilage matrix, where aggrecan is the most common GAG-containing protein in cartilage. Therefore, it may be possible that orally administered HA facilitates the accumulation of GAG within cartilage caused by the exercise. This possibility may be demonstrated by the comparison of subjects between the HA and placebo groups by means of MRI. We think this will be an interesting subject to study. Hopefully, the results of this study may provide some clues to understanding why HA can alleviate the symptoms of knee OA. 

Thus, despite several limitations, the results of the present study have shown that the oral administration of HA may have beneficial therapeutic effects on patients with symptomatic knee OA and may be even more beneficial for relatively young patients. We consider that our results may well be of some help to both people suffering from knee OA and those who take care of them. 

The effect of HA administration on the symptoms of knee OA was investigated in a one-year, prospective randomised study. The results of the study indicated that the treatment is effective when combined with quadriceps strengthening exercise. This effect of HA administration may be more obvious if a subject is 70 years old or younger. 

## Figures and Tables

**Table 1:** Demographic and clinical characteristics of subjects at enrollment.

	HA	Placebo
Number of subjects	30	30
Male	7	7
Female	23	23
Age	69 ± 1	69 ± 1
JKOM score		
Total	43 ± 2	46 ± 3
Subscales		
Pain and stiffness in knees	15 ± 1	16 ± 1
Condition in daily life	16 ± 1	17 ± 1
General activities	8 ± 1	9 ± 1
Health conditions	4 ± 0	4 ± 0
Kellgren and Lawrence grade		
Grade 2	13	13
Grade 3	17	17
Additional medication		
NSAID, oral administration	8	9
NSAID, external use	16	15
HA, intra-articular injection	23	28

Results are mean ± SE.

**Table 2:** Change of JKOM score during study period.

		Time (month)				
		0	2	4	6	12
Total	HA	100.0 ± 0.0	80.6 ± 3.1*	71.8 ± 3.0*	72.4 ± 3.5*	70.9 ± 3.9*
	Placebo	100.0 ± 0.0	87.4 ± 3.1*	78.9 ± 3.1*	74.8 ± 3.2*	72.1 ± 4.0*
Subscale						
Pain and stiffness in knees	HA	100.0 ± 0.0	77.0 ± 3.6*	70.2 ± 3.7*	73.7 ± 4.2*	66.8 ± 4.4*
	Placebo	100.0 ± 0.0	84.6 ± 4.6*	76.5 ± 3.9*	71.8 ± 4.0*	72.5 ± 8.0*
Condition in daily life	HA	100.0 ± 0.0	83.7 ± 3.8*	77.6 ± 3.9*	77.0 ± 4.1*	76.2 ± 4.4*
	Placebo	100.0 ± 0.0	89.2 ± 3.7*	83.9 ± 4.1*	81.3 ± 3.5*	76.0 ± 3.4*
General activities	HA	100.0 ± 0.0	94.2 ± 7.4	75.5 ± 4.8*	73.3 ± 5.0*	81.1 ± 5.8*
	Placebo	100.0 ± 0.0	92.5 ± 4.9	80.3 ± 5.8*	73.4 ± 4.8*	72.8 ± 4.1*
Health condition	HA	100.0 ± 0.0	74.0 ± 6.0^∗#^	64.6 ± 7.7^∗#^	63.0 ± 6.1^∗#^	60.9 ± 5.6^∗#^
	Placebo	100.0 ± 0.0	96.1 ± 8.2	91.1 ± 9.9	88.3 ± 10.1*	81.2 ± 6.0*

Results are shown by relative rations (in percent) against scores at baseline. Mean ± SE of 18 (HA group) or 20 (placebo group) subjects are shown.

^∗^ *P* < 0.05 against baseline; ^#^ *P*< 0.05 against placebo group.

**Table 3:** Kellgren and Lawrence grade before and 12 months after administration.

		Time (month)	
		0	12
HA	Grade 2	9	8
	Grade 3	9	10
Placebo	Grade 2	9	9
	Grade 3	11	11

Numbers of subjects being Kellgren and Lawrence grade are shown.

**Table 4:** Use of additional medication.

		Time (month)				
		0	2	4	6	12
Medication						
NSAID, oral administration	HA	9	8	7	7	5
	Placebo	10	10	7	7	4
NSAID, external use	HA	16	15	15	13	13
	Placebo	18	17	17	14	13
HA, intra-articular injection	HA	6	7	8	7	5
	Placebo	8	6	7	7	6

Numbers of subjects requiring respective treatments are shown.

NSAID: nonsteroidal anti-inflammatory drugs.
